# Emotion and Implicit Timing

**DOI:** 10.1371/journal.pone.0158474

**Published:** 2016-07-05

**Authors:** Sylvie Droit-Volet

**Affiliations:** Université Clermont Auvergne, Laboratoire de psychologie sociale et cognitive, CNRS, UMR 6024, Clermont-Ferrand, France; University of Udine, ITALY

## Abstract

This study examined the effects of emotion on implicit timing. In the implicit timing task used, the participants did not receive any temporal instructions. Instead they were simply asked and trained to press a key as quickly as possible after a stimulus (response stimulus) that was separated from a preceding stimulus by a given temporal interval (reference interval duration). However, in the testing phase, the interval duration was the reference interval duration or a shorter or longer interval duration. In addition, the participants attended two sessions: a first baseline session in which no stimulus was presented during the inter-stimulus intervals, and a second emotional session in which emotional facial expressions (angry, neutral and sad facial expressions) were presented during these intervals. Results showed faster RTs for interval durations close to the reference duration in both the baseline and the emotional conditions and yielded a U-shaped curve. This suggests that implicit processing of time persists in emotional contexts. In addition, the RT was faster for the facial expressions of anger than for those of neutrality and sadness. However, the U-shaped RT curve did not peak clearly at a shorter interval duration for the angry than for the other facial expressions. This lack of time distortion in an implicit timing task in response to arousing emotional stimuli questions the idea of an automatic speeding-up of the interval clock system involved in the representation of time.

## Introduction

Subjective time is distorted under the effect of emotion. For example, time seems to speed up in dangerous situations when a person threatens to attack us. This effect of emotion on the judgment of time has been extensively studied in recent decades [1]. In a seminal study conducted in our laboratory, participants were presented with pictures of faces expressing different emotions, namely that of anger as well as of happiness, sadness and neutrality [2]. Their task was to judge whether the duration of presentation of these facial expressions was more similar to a short or long anchor duration previously learned in a non-emotional context (temporal bisection task). Results showed that, compared to other facial expressions, the participants more often associated the angry faces with the long than with the short standard duration. This temporal lengthening effect in response to angry faces has since been replicated with different negative high-arousing emotional stimuli (e.g., affective pictures, sounds, electric shocks) [3,4,5,6,7]. This emotional effect on time judgment has generally been explained as resulting from the increase in the physiological arousal level that automatically speeds up the internal clock system that provides the raw material for time representation [8]. According to the internal clock model [9,10], a clock system emits pulses (ticks, oscillations) that are gated into an accumulator during the stimulus to be timed, with the result that subjective time depends on the number of pulses accumulated. When the clock runs faster, more pulses are accumulated and the stimulus duration is judged longer. Dopamine activity in the cortico-striatal circuits has been considered to be the major factor controlling the speed of the internal clock [11]. Since it is well known that the emotion of fear increases the level of dopamine in the brain [12,13], it is likely that the perception of threatening stimuli accelerates the clock system involved in time representation. However, since the effects of emotion on behavior are particularly complex [14], the question of the different mechanisms involved in time judgment in emotional states remains a matter of debate today. In human beings, conscious processes may indeed influence time judgment, for example the decision to respond long or short in a bisection task. Recently, Droit-Volet et al. [15] demonstrated that explicit knowledge about subjective time distortion modulates the effects of emotion on time judgment. To further examine whether emotional effects on judgments of durations are due to an automatic speeding-up of the internal clock system, we decided to test emotional effects on implicit processing of time in the present study.

In explicit time judgment tasks, such as the temporal generalization task, participants are taught that they must learn a reference duration. They are then overtly instructed to compare this duration with probe durations (shorter, longer or equal). In this way, they are made consciously aware of temporal aspects of the task and are required to process time intentionally. Unlike in explicit time judgment tasks, the participants in implicit timing tasks are unaware that they are processing time [16,17,18]. For example, participants can be presented with two stimuli separated by an interval, but without being given any temporal instructions. They are simply told to press as quickly as possible after the second stimulus (response signal). In this condition, the critical measure is the reaction time (RT), i.e. the minimum time needed to respond to a stimulus. It has been demonstrated that RT is influenced by the interval between two stimuli [19]. RT is indeed faster for stimuli presented after a long rather than a short interval (foreperiod effect), at least when foreperiods are variable and have a uniform probability of presentation. This effect is explained by the increasing conditional probability over time that the target stimulus is going to appear, given that it has not already occurred (the hazard function) [20,21,22]. This would lead to the automatic processing of the time context and make it possible to predict and anticipate incoming events. However, in most “foreperiod” studies, participants are not familiarized with a specific time of stimulus occurrence that would allow them to accurately predict the time window in which the stimulus is expected to occur. In a recent study based on a paradigm of temporal discrimination (temporal generalization), Piras and Coull [23] trained their participants to press a button as quickly as possible after a second stimulus that was separated from the first by a given inter-stimulus interval (reference interval duration). The authors' idea was that participants implicitly construct their temporal expectation for the response stimulus through the automatic learning of this reference interval duration. In the subsequent testing phase, different interval durations were presented, including the reference interval and other intervals that were shorter or longer than it. The results showed that RT varied as a function of interval durations. They was indeed faster during a time window close to the reference interval duration, and increased outside this temporal window for interval durations shorter or longer than the reference interval, thus resulting in a U-shaped curve. It seems quite unlikely that participants are conscious of the rules governing their reaction times. The variation in RT as a function of interval durations has therefore been explained in terms of time processing that operates largely independently of consciousness [21,23,24].

The aim of the present study was therefore to examine the effect of an emotional context on RT in a paradigm similar to that used by Piras and Coull [23]. The participants were therefore trained to press the “0” key of the computer keypad as quickly as possible after a stimulus, which was separated from a preceding stimulus by a given interval (reference interval). A relatively high number of training trials were used to ensure a stable representation of the reference interval in memory. In the testing phase, the participants’ task was the same. However, probe inter-stimulus intervals were tested, involving the presentation of the reference interval as well as of intervals shorter and longer than it. This implicit timing task was performed in a non-emotional context (with no stimulus presented during the interval duration) in an initial session, and in an emotional context in a second session. In this second session, emotional pictures were presented during the inter-stimulus interval. Given that emotional effects on time judgment are known to occur in response to emotional facial expressions, we decided to use facial expressions of anger and neutrality as our emotional stimuli. Our hypothesis was that RT should be faster for the probe interval durations close to the reference interval. However, in an emotional context, if the perception of angry faces increases the speed of the interval clock, the RTs should shorten earlier, i.e. at probe interval durations shorter than the reference interval.

## Experiment 1

### 2.1 Method

#### 2.1.1 Participants

Forty-two undergraduate psychology students from Clermont Auvergne University (*Mean age* = 19.48; *SD* = 1.10; 32 females and 8 males) participated in this experiment. The students signed a written informed consent before their participation to this experiment. This experiment has been conducted according to the principles expressed in the declaration of Helsinki. The procedure used was approved by the Sud-Est VI Statutory Ethics Committee (CPP, France). The students received course credits in exchange for their participation.

#### 2.1.2 Material

The participants were seated in a quiet room in the laboratory of the psychology department facing a computer that delivered and recorded all experimental events using E-prime (Psychology Software Tools Inc.). They responded by pressing the “0” key of the computer keypad with the index finger of the dominant hand. The stimulus at onset and offset of the temporal interval was a 100-ms auditory stimulus. The emotional stimuli were six photographs of 3 different women expressing anger or neutrality. These faces were taken from a validated set of facial expressions [25].

#### 2.1.3 Procedure

The participants (21 per group) were assigned to either a short or a long duration group. One participant was nevertheless excluded from the final sample because he always produced RT longer than 1.5 s. For the short group, the reference interval duration was 500 ms and the probe interval durations were 200, 300, 400, 500, 600, 700 and 800 ms. For the long group, the reference duration was 1000 ms and the probe durations 400, 600, 800, 1000, 1200, 1400 and 1600 ms. Each participant completed two successive sessions (baseline session and emotion session) consisting of a training phase and a testing phase. In each phase, the participant initiated a trial by pressing on the spacebar of the computer keyboard after the word “ready/prêt” was presented in the center of the computer screen. Two auditory signals were delivered 200 ms after trial initiation, with an inter-signal interval corresponding to either the reference or probe duration. The inter-trial interval was randomly selected from a continuous range between 500 and 1000 ms.

In the training phase (20 trials), the inter-signal interval always corresponded to the reference duration, thus ensuring a stable reference memory. The participants did not receive any temporal instructions and were only asked to press as quickly as possible after hearing the second signal. In the testing phase, they were told that they had successfully learnt to press quickly after the second auditory signal, and that they must now continue to do so. In this phase, the reference duration was presented 9 times (9 trials) and the other probe durations 3 times each (6 x 3 trials). The trial presentation order was randomized within each trial block (3 blocks of 9 trials). The training phases were the same for the baseline and the emotion session. The only difference between the two sessions lay in the testing phase. In the baseline testing phase, there was a total of 27 trials (9 + 18). In the emotion testing phase, an emotional stimulus was presented during the interval between each auditory signal. As there were two emotional stimuli (angry and neutral facial expressions), this made a total of 54 trials (27 trials x 2 emotional stimuli), with the emotional stimuli being randomly presented within each block (3 blocks of 9 x 2 trials).

### 2.2 Results and discussion

**Fig 1:**
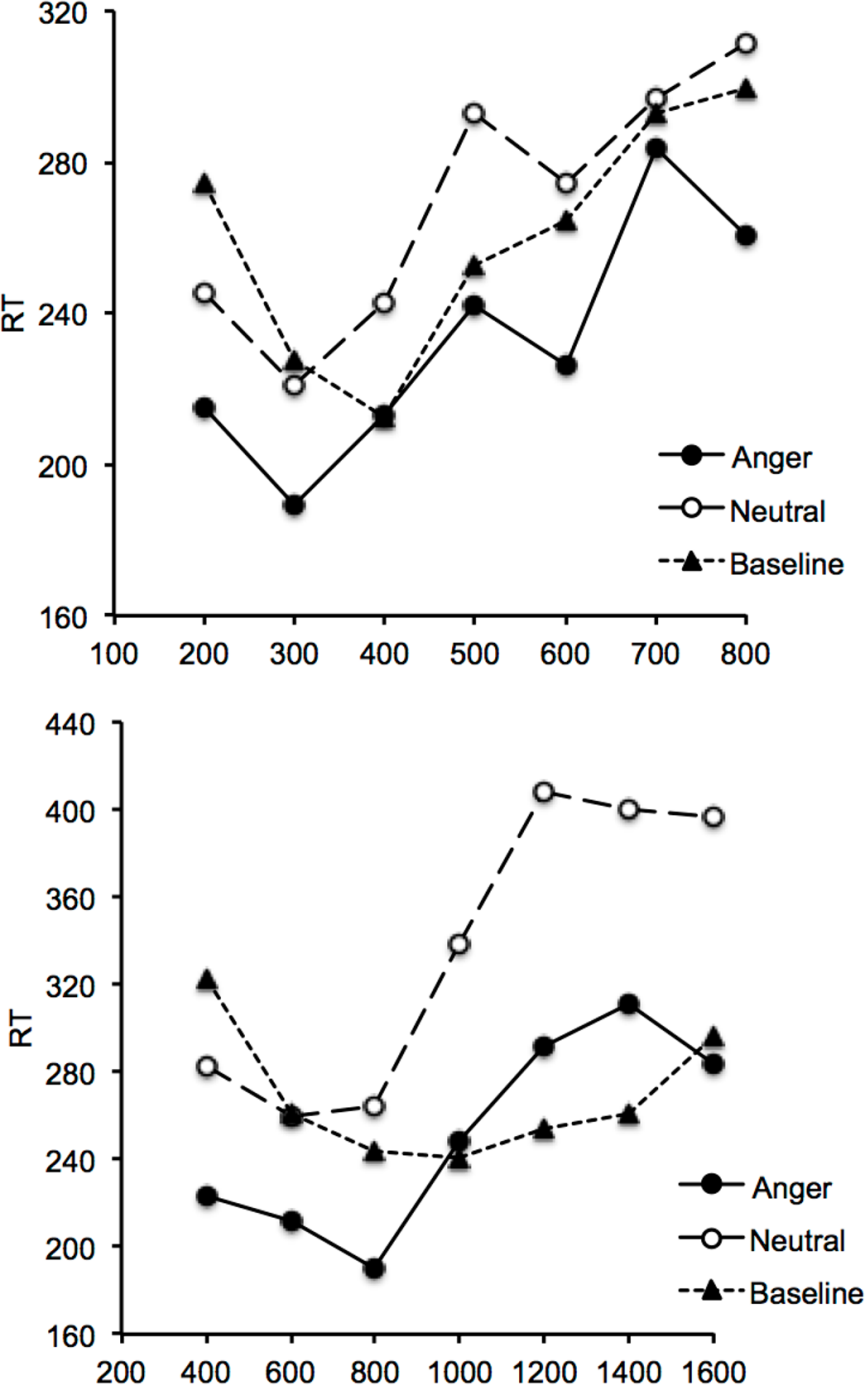
Reaction time and interval duration for two duration ranges. Reaction time plotted against interval duration in the baseline condition and the emotion condition (anger and neutral) for the short (200/800) and the longer duration (400/1600) group.

**Fig 2:**
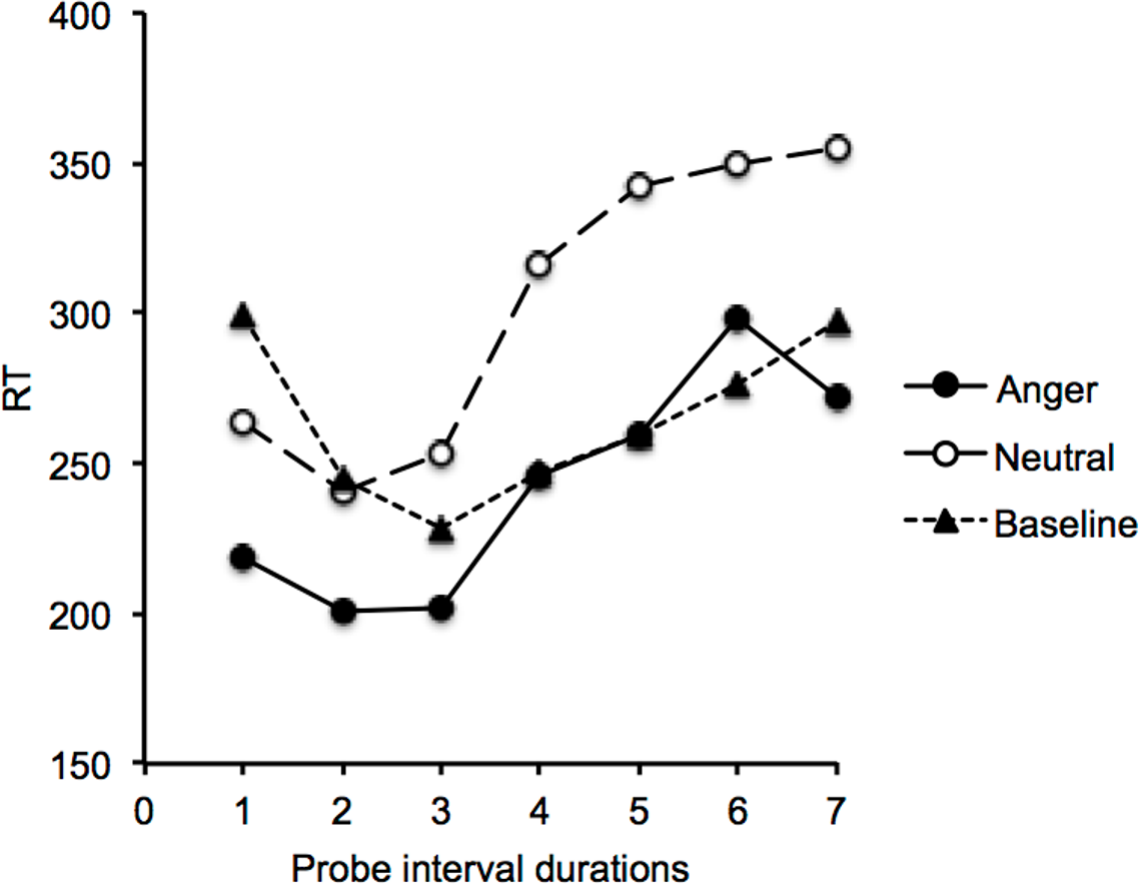
Reaction time and interval duration. Reaction time plotted against interval duration (duration group averaged) in the baseline condition and the emotion condition (anger and neutral).

Fig 1 shows Reaction Time (RT) plotted against interval duration in the short and the long duration range condition when either no stimulus or an emotional stimulus (angry vs. neutral faces) was presented during the interval (Figure A in S1 File). The RT curves were U-shaped in all conditions, with RT decreasing to a minimum value before subsequently increasing again. For the baseline session, the ANOVA performed on RT with one within-subjects factor (interval duration) and one between-subjects factor (duration range) confirmed the existence of a main effect of interval duration, *F*(6, 234) = 5.01, *p* = .0001, *η*^*2*^_*p*_ = .11. The main effect of duration range, *F*(1, 39) = 0.03, *p* = .87, and the duration interval x duration range interaction, *F*(6, 234) = 1.45, *p* = .20, were not significant. The significant effect of interval duration indicated that RT was affected by the temporal interval before the response signal, i.e. a finding that is consistent with the implicit processing of time. As suggested by Fig 2, in which the two duration ranges are averaged, the minimum value of the U-curve (peak time) in the baseline session was located at the probe interval duration just shorter than the reference interval duration (D3). The RT was indeed shorter for D3 than for the shortest (D1) or the two longest interval durations (D6, D7) (Bonferroni tests, *p* < .05), while no significant difference was observed in RT between these anchor durations (*p* > .05).

For the emotion session, the ANOVA performed on RT also found a significant main effect of interval duration, *F*(6, 234) = 12.53, *p* = .0001, *η*^*2*^_*p*_ = .24, confirming that RT continued to be affected by the temporal interval before the response signal in an emotion context. The significant main effect of interval duration was verified in all emotional conditions (short interval duration range: angry faces, *F*(6, 114) = 3.73, *p* = .0001, *η*^*2*^_*p*_ = .29; neutral faces, *F*(6, 114) = 2.5, *p* = .026, *η*^*2*^_*p*_ = .12; long interval duration range: angry faces, *F*(6, 120) = 4.44, *p* = .001, *η*^*2*^_*p*_ = .18; neutral faces, *F*(6, 120) = 5.28, *p* = .001, *η*^*2*^_*p*_ = .21). The main effect of emotion, *F*(1, 39) = 44.62, *p* = .0001, *η*^*2*^_*p*_ = .53, and the duration range x emotion interaction, *F*(1, 39) = 7.08, *p* = .01, *η*^*2*^_*p*_ = .15, were also significant. The main effect of duration range, *F*(1, 39) = 3.20, *p* = .08, and the other interactions with this factor did not reach significance (*p* > .05). The main effect of emotion indicated shorter RT with the emotional stimulus of anger (*M* = 242.17, *ES* = 11.50) than with the emotional stimulus of neutrality (*M* = 302.29, *ES* = 13.77). The magnitude of the difference in RT between these two emotional stimuli was nevertheless higher for the long than for the shorter duration range (84.06 vs. 36.17, *F*(1, 39) = 7.08, *p* = .01).

The participants therefore always responded faster in response to the facial expression of anger than to the neutral facial expression. The emotion x interval duration interaction, *F*(6, 234) = 0.79, *p* = .58, did not reach significance. The minimum value of the U-curve (peak time) occurred indeed earlier in the two emotion conditions than in the baseline condition without stimulus, i.e., at D2 rather than D3 (Fig 2). However, the minimum value of RT appeared at the same probe interval duration (D2) for anger and neutral emotion. Therefore, with the procedure used, there was no clear difference in the implicit processing of time between the two emotional stimuli tested (anger vs. neutrality). Based on our a priori hypothesis [26], we nevertheless decided to run additional analyses and found only that the significant increase in RT after this D2 value emerged earlier for anger than for neutrality. Indeed, RT were significantly longer for D4 than for D3 in response to anger (Bonferroni test, *p* < .05), while this significance difference in RT was only observed between D6 and D3 when a neutral facial expression was presented.

**Fig 3:**
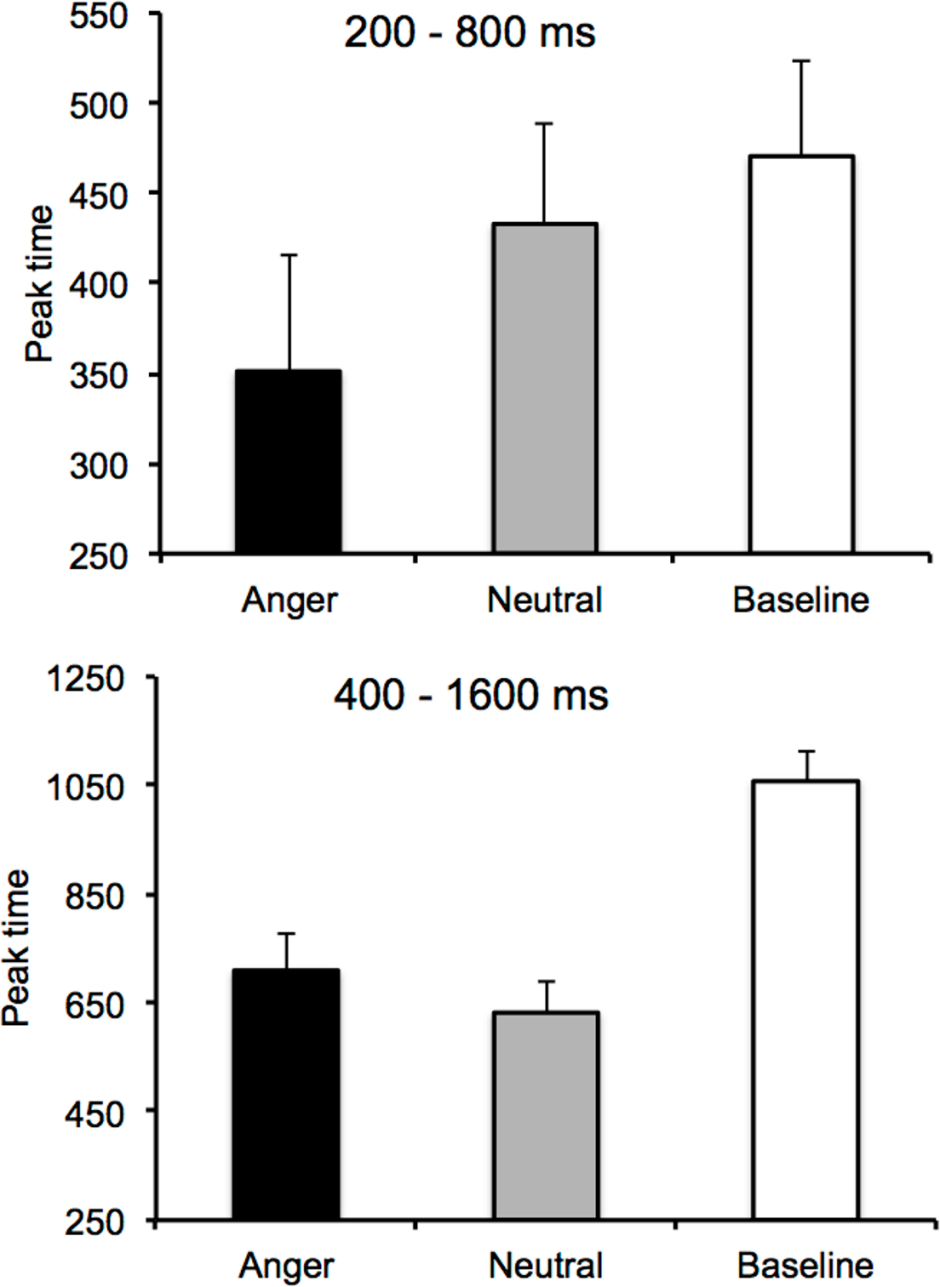
Peak time for two duration ranges. Peak time for the U-shaped reaction time curve in the baseline condition and the emotion condition (anger and neutral) for the short (200/800) and the longer duration (400/1600) group.

To further examine the value of the peak time (minimum value) of the U-shaped curves, we decided to fit each individual curve with the polynomial function from the GraphPrism program. The polynomial fit procedure provided reasonably good fits of the temporal curves for most of the participants (*p* < .05). However, the fit was not significant for some participants. This was probably due to the small number of trials we initially used in each condition in order to avoid a fall-off in the short-lived emotion effect. In such cases, we simply took the probe duration corresponding to the lowest RT value. This procedure was not possible for 4 participants who produced low RT at different probe durations, and who were therefore excluded from the following statistical analyses. Fig 3 showed the peak time in the baseline and the emotion session (Figure B in S1 File). In the emotion session, the effect of emotion was significant in the short duration condition, *F*(1, 19) = 4.29, *p* = .05, *η*^*2*^_*p*_ = .18, but not in the long duration condition, *F*(1, 19) = 2.47, *p* = .13. This finding therefore indicated that the peak time was lower for the angry than for the neutral emotion, but only in the short duration range condition. This suggests a leftward shift of the U-shaped curve, which was greater for the angry than for the neutral emotion when the inter-signal intervals were short (< 800 ms). The comparison between the peak time for the different emotion conditions and the baseline condition revealed that the peak time was systematically higher for the baseline condition without stimulus than for the two emotional stimuli presented during the temporal interval (all *p* < .05), with the exception of the difference in the peak time between the neutral emotional stimulus and the no-stimulus condition for the short duration range, which did not reach significance (*F*(1, 18) = .50, *p* = .49).

In sum, our results show that RT were systematically affected by the nature of the stimulus presented during the temporal interval between two signals, being faster for the high-arousing emotional stimulus (angry expression) than for the neutral emotional stimulus or in cases where no stimulus was administered. They also show that RT were affected by the temporal interval between the two signals, forming a U-shaped curve in all the experimental conditions, whether emotional or not. This suggests the existence of an implicit processing of time in an emotional context as well as in a non-emotional context when no stimulus was presented during the inter-signal interval. However, the U-shaped curve of RT, depending on inter-signal interval, did not clearly change as a function of emotions. Indeed, the U-shaped RT curves were shifted toward the left, with a lower peak time, in the condition with pictures of facial expressions compared to the baseline condition without stimulus. However, this leftward shifting did not appear to be significantly more marked for the angry faces than for the neutral facial expressions. Indeed, the emotion x interval duration interaction did not reach significance. The peak time of the U-curve nevertheless tended to be lower for the angry than for the neutral emotion, but only in the short duration range condition. This suggests that the RT for the response signal after the inter-signal interval was more impacted by the presentation of facial expressions in the short than in the long duration range condition. This may be related to action preparation processes that occur earlier with long inter-signal intervals than with shorter ones [20,21,22], thereby limiting the examination of emotional effects in a “foreperiod” paradigm to short interval durations. We initially assumed that an automatic speeding up of the internal clock in the high-arousal emotion condition (angry face) would affect the RT in both the short and the long duration range. Our results were not consistent with this hypothesis and therefore suggest that, in our paradigm, additional processes have probably interfered with the timing of action preparation during the temporal interval between the warning and the response signal. Indeed, the results in Experiment 1 showed that the difference in RT for the angry and neutral emotions was very marked, but that the shifting of the U-shaped curve toward shorter interval durations for anger compared to neutrality remained weakly significant. This is likely to be due to the general context in the emotional session that produced within-trials interference effects on RT. We therefore decided to run a second experiment to examine the short duration range condition in two different emotional sessions (groups). Facial expressions of anger and neutrality were presented to one group of participants in one and the same session, whereas facial expressions of sadness and neutrality were presented to the other group. Sad facial expressions were chosen because, while they are also unpleasant pictures (negative emotion), they are less arousing than angry faces that represent a more imminent danger (attack) [27]. In addition, studies of explicit time judgments have not found any time distortion with this emotion [8].

## Experiment 2

### 3.1 Method

#### 3.1.1 Participants

Forty new undergraduate psychology students participated in this study conducted according to the principles expressed in the declaration of Helsinki (*Mean age* = 19.9 years, *SD* = 0.99, 31 females and 9 males). They received course credits for their participation. They also signed written informed consent before their participation. The procedure of Experiment 2 was approved by the Sud-Est VI Statutory Ethics Committee (CPP, France).

#### 3.1.2 Material and procedure

The material and procedure were exactly the same as those used in Experiment 1, except for the facial expression of sadness which were taken from Ekman and Friesen’s battery of facial expressions [25]. In addition, in order to increase the emotion effect, the emotional stimuli were presented 500 ms before the first auditory signal and continued during the temporal interval between the two auditory signals. The participants’ task was still to press as quickly as possible after the second signal. In Experiment 2, they were assigned to an “anger” or “sadness” group (20 participants per group). Each group took part in two successive sessions: the baseline session and the emotional session. The training and the testing phases for these two sessions were similar between the groups, except for the emotional stimuli presented in the testing phase of the second session: angry and neutral facial expressions in the anger group and sad and neutral facial expressions in the sadness group.

#### 3.2.3 Results

**Fig 4:**
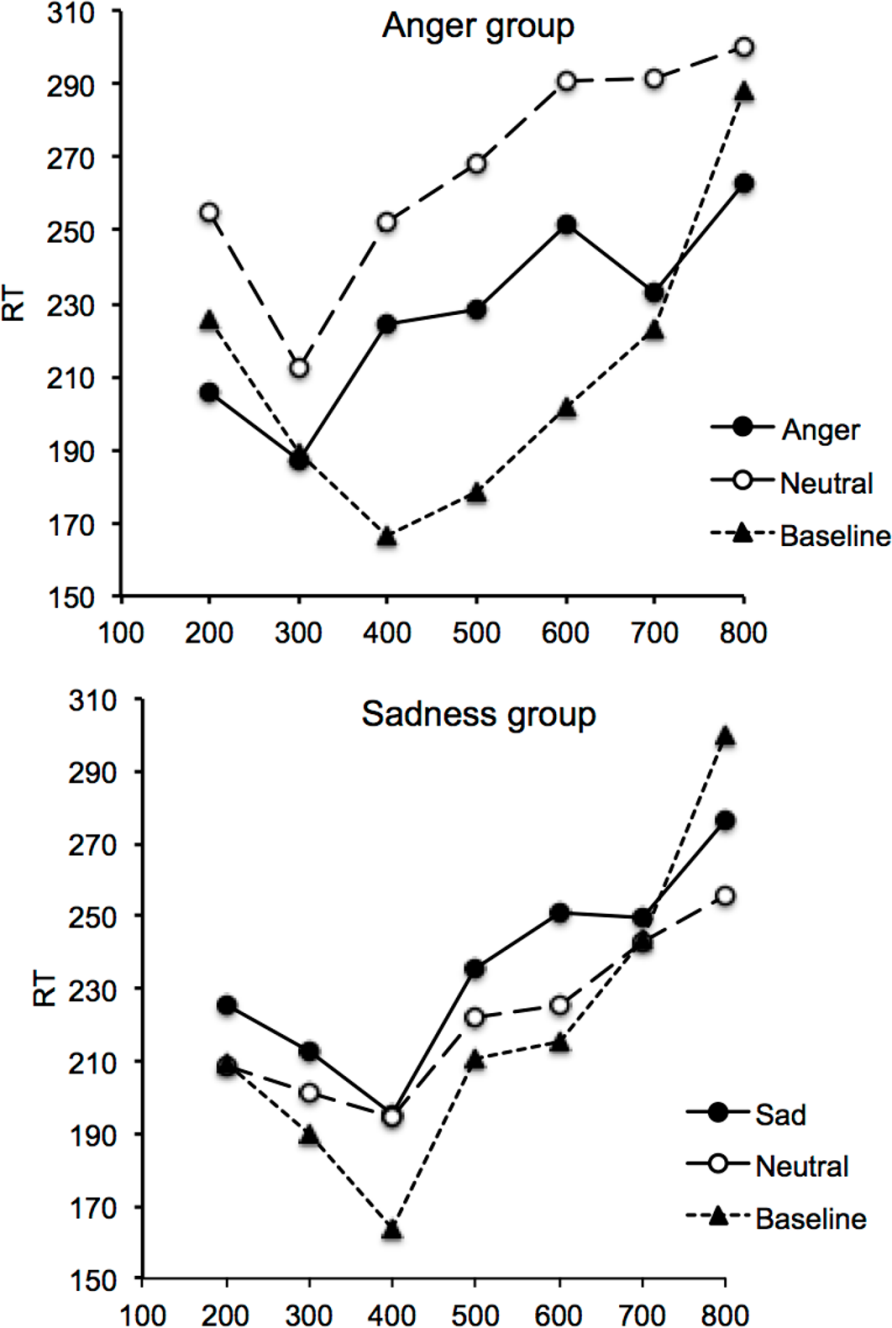
Reaction time and interval duration for the anger and the sadness group. Reaction time plotted against interval duration in the baseline line and the emotion condition for the “anger” group (anger vs. neutral) and the “sadness” group (sadness vs. neutral) group.

Fig 4 shows RT as a function of probe interval durations for the session without stimulus and the second session with emotional stimuli in the anger and sadness groups (Figure C in S1 File). Unlike in Experiment 1, RT appeared to be longer with than without emotional pictures. This is probably due to the fact that the pictures were presented both during the interval duration and before the first signal of 500 ms. The RT curve was nevertheless U-shaped in all conditions, thus confirming the implicit processing of time between these two auditory signals.

The ANOVA conducted on RT for the baseline session with the group and the duration interval as factors again found a significant main effect of interval duration, *F*(6, 228) = 12.66, *p* = .0001, *η*^*2*^_*p*_ = .25, indicating variations of RT as a function of interval before the response signal. As easy to predict, the effect of group, *F*(1, 38) = 0.15, *p* = .71, and the group x interval duration, *F*(6, 228) = 0.66, *p* = .69, were not significant.

For the session with the emotional stimuli, the ANOVA performed on RT with 3 factors (group, emotion, interval duration) also showed a significant main effect of interval duration, *F*(6, 228) = 8.59, *p* = .0001, *η*^*2*^_*p*_ = .18. The significant main effect of interval duration was verified in all emotional conditions (anger group: angry faces, *F*(6, 114) = 3.77, *p* = .002, *η*^*2*^_*p*_ = .17; neutral faces, *F*(6, 114) = 3.41, *p* = .004, *η*^*2*^_*p*_ = .15; sadness group: sad faces, *F*(6, 114) = 2.45, *p* = .02, *η*^*2*^_*p*_ = .11; neutral faces, *F*(6, 114) = 2.67, *p* = .02, *η*^*2*^_*p*_ = .12). RT were therefore influenced by the interval duration between the two auditory signals in all emotional conditions, as well as in the baseline condition. However, there was also a significant main effect of emotion, *F*(1, 38) = 6.24, *p* = .02, *η*^*2*^_*p*_ = .14, as well as a significant emotion x group interaction, *F*(1, 38) = 19.42, *p* = .0001, *η*^*2*^_*p*_ = .34. The 3-way interaction (emotion x group x duration interval) did not reach significance, *F*(6, 228) = 0.28, *p* = .95.

When each group was taken separately, neither an effect of emotion, *F*(1, 19) = 1.65, *p* = .21, nor an interaction between emotion and interval duration, *F*(6, 114) = 0.49, *p* = .81, were found for the sadness group. RTs were therefore similar between the sad emotional stimulus (*M* = 232.52) and the neutral emotional stimulus (*M* = 221.58). Post-hoc comparisons of RTs between the sad and the neutral faces did not show any significant difference for any of the interval durations (all *p* > .05). In the sadness group, RT therefore varied with interval duration but not as a function of the emotional stimuli presented during the inter-signal interval. By contrast, in the anger group, the effect of emotion reached significance, *F*(1, 19) = 26.62, *p* = .0001, *η*^*2*^_*p*_ = .58. Indeed, RT were faster in response to anger (*M* = 227.56) than to the neutral emotion (*M* = 267.17). However, the emotion x interval duration interaction did not reach significance, *F*(6, 114) = 0.34, *p* = .91, confirming the lack of temporal modulation of the emotional effect on RT when the angry and the neutral faces were presented in the same session. However, based on our a priori hypotheses [26], we performed additional analyses to compare emotion-related differences in RT for the different interval durations. There was no effect of emotion for the three shortest probe interval durations (200 ms, *F*(1, 19) = 2.56, *p* = .11; 300 ms, *F*(1, 19) = 2.05, *p* = .17; 400 ms, *F*(1, 19) = 2.54, *p* = .13) or the longest probe duration (800-ms, *F*(1, 19) = 3.25, *p* = .09), whereas the effect of emotion was observed at the intermediate interval durations of 500, 600 and 700 ms (*F*(1, 19) = 16.55; *F*(1, 19) = 4.32; *F*(2, 38) = 7.16, respectively, all *p* < .05). RT were thus shorter for the angry than the neutral faces at the interval durations close to the reference interval durations. These last results thus suggested that the anger-neutral difference in RT tended to change across the probe interval durations.

**Fig 5:**
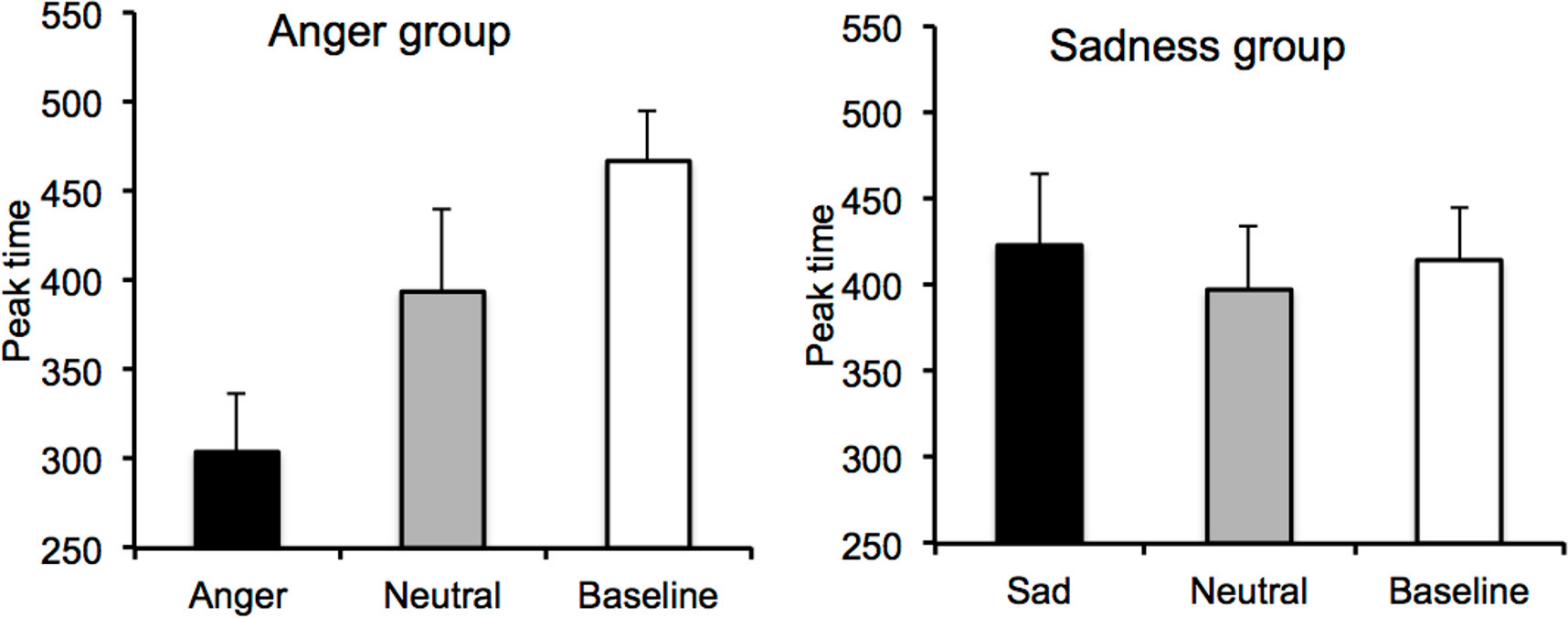
Peak time for the anger and the sadness group. Peak time for the U-shaped reaction time curve in the baseline condition and the emotion condition for the “anger” group (anger vs. neutral) and the “sadness” group (sadness vs. neutral) group.

As in Experiment 1, we also measured the peak time (minimum value) using the same procedure as described above (Fig 5 and Figure D in S1 File). The overall analysis of variance on peak time for the emotion session including the sadness and the angry group showed that the emotion x group did not reach significance (*F*(1, 38) = 2.73, *p* = .10). This interaction was again not significant. Only pair-wise comparisons of peak time between each emotion condition revealed some significant trends. Indeed, these comparisons suggested that the RT curve peaked at a shorter interval duration for the angry (*M* = 303.92) than for the sad faces (*M* = 422.98), *F*(1, 38) = 5.19, *p* = .03, whereas the peak time was similar for the neutral faces between the angry and sadness groups, *F*(1, 38) = .004, *p* = .95. Indeed, for the sadness group, the peak time was close to the probe interval duration value just shorter than the reference duration in all emotional conditions as well as in the baseline condition (*M*_*sad*_ = 422.98; *M*_*neutral*_ = 397.27; *M*_*no-stimulus*_ = 414.41). There was indeed no difference in the peak time between the sad and the neutral faces, *F*(1, 19) = .23, *p* = .64. By contrast, for the anger group, the RT curve peaked at a shorter interval duration value in response to the anger stimulus (*M* = 303.93, *SE* = 32.88) than it did in the presence of the neutral stimuli (*M* = 393.69, *SE* = 45.71), *F*(1, 19) = 4.15, *p* = .04.

## General Discussion

This study examined the effects of emotion (facial expressions) on implicit timing. In two experiments, participants were trained on a reference interval between two auditory signals, followed by a testing phase that included both the reference interval and different probe durations that were either shorter or longer than the reference interval. The participants did not receive any temporal instructions. They were simply told to press a key as quickly as possible after the second signal. Our results showed that the speed of RT depended on the inter-signal interval duration. RT were indeed faster for interval durations close to the reference duration than for the other shorter or longer durations and formed a U-shaped curve. This finding is consistent with the results reported by Piras and Coull [23] using a similar procedure, as well as by other authors working within the “foreperiod” paradigm [17,18,28]. There is currently a debate as to whether the foreperiod phenomena are purely automatic or not [23]. Some authors consider that the variation in RT as a function of the time period preceding the response signal reflects an automatic processing of the inter-signal interval, which permits subjects to anticipate the arrival of the next signal and to be prepared to respond as quickly as possible when it appears [23]. However, recent experimental evidence has shown that the foreperiod function is flatter in a dual-task than in a single-task condition, thus suggesting the activity of cognitive control processes during the temporal preparation for action in the foreperiod paradigm [29,30]. Although the processes involved in the implicit timing task are complex and are probably not completely automatic, we can nevertheless assume that the processing of time requires less cognitive resources and operates more automatically in this task than in an explicit timing task [31]. Consequently, our results depicting the U-shape of RT as a function of the interval duration between the warning and the response signal suggest the existence of an implicit processing of time that affects motor preparation processes. However, our results also demonstrate that this implicit processing of time persists in an emotional context. Indeed, the U-shaped RT curves were obtained in all the experimental conditions used in our study, i.e. in the baseline session without stimuli presented during the inter-signal interval, and in the emotion session with different emotional facial expressions (anger, sadness, neutrality). Although they did not significantly affect our results, two possible limitations to our study must nevertheless be pointed out as they may be relevant for future experiments. The first is related to the low number of trials used in our study in order to take account of the short-lived emotional effect. In some of the subjects, this produced a U-shaped curve that did not fit well with a polynomial function. The second is related to the emotion session, which was administered after a first non-emotion session and may have induced some learning-related improvement in reaction times, thus limiting emotion effects on timing. This approach was adopted in order to ensure a stable memory representation of the reference interval duration. Whatever the case may be, our data clearly demonstrate that the implicit processing of time persists in different emotional contexts despite these experimental conditions.

Our data also showed that the emotional context affected the RT in the implicit timing task used in our study. Indeed, independently of the value of the inter-signal intervals, the RT were always faster when the emotional image presented during this inter-signal interval was that of an angry face compared to a neutral or sad face. This result is entirely consistent with the results of numerous studies showing that high-arousing emotions speed up most motor responses (simple reaction time, gait, etc.) [32,33]. When emotional stimuli, such as threatening facial expressions, constitute a signal relevant for the defensive system, they trigger fast and appropriate action (withdrawal, escape, attack) [34,35,36]. The survival instinct thus activates the process of preparation for action and makes it possible to produce rapid action in the event of potential danger [37,38]. In sum, the processing of threatening stimuli, such as the facial expression of anger used in our study, is prioritized in order to permit a rapid response [39,40,41]. Our results support this explanation by showing that RT was faster for angry faces than for other facial expressions (neutral and sad faces) or in the case where no stimuli were presented during the inter-signal interval. Our data therefore suggest an acceleration of the executive motor functions in response to angry faces compared to other faces in an implicit timing task using RT as measure.

However, the aim of our study was to examine whether this acceleration of RT with angry faces also varied with the inter-signal duration, i.e. occurring at shorter probe interval durations. This would indicate that the perception of angry faces affects not only the motor processes involved in action preparation, but also the internal clock system underlying the timing of action. As explained in the introduction, when the internal clock speeds up, more time units (pulses, oscillations) are accumulated, and time production is shorter [8]. In our implicit time task, this clock-effect should have produced a leftward shift of the U-shaped RT curve for angry faces compared to other faces. However, the general analyses of variance in our experiments did not provide convincing results, given that no significant interaction between emotions and interval durations was found. Consequently, contrary to our hypothesis, the shortening of RT did not occur earlier for angry faces than for the other facial expressions. Only additional pair-wise comparisons suggested a tendency for a temporal modulation of the peak time of the U-curve as a function of emotions. Indeed, in the baseline session, the RT curve peaked at an interval duration close to the reference interval duration (400 ms). In the emotional session with facial expressions of sadness, the RT curve also peaked at 400 ms, and no difference in the peak time was observed between the sad and neutral facial expressions. By contrast, in the emotional session with angry facial expressions, the RT curve peaked at a shorter interval duration, i.e., at 300 ms, such that the peak time was lower with the angry than with the sad faces. Furthermore, the RT curve also peaked at a shorter duration interval for the angry faces than for the neutral face in our experiments, but only in the 200-800-ms duration range. Whatever the case may be, for the implicit timing task used, these statistical results did not provide a clear demonstration of an emotion-dependent modulation of RT as a function of inter-signal interval.

The question raised is: why did we not find results providing a clearly demonstration of a temporal variation of RT as a function of emotion in our implicit timing task? As reported in the introduction, a dilation of time in high-arousing emotional contexts has been clearly established in explicit time judgment tasks used in human adults [1,8]. A fundamental difference between the explicit and the implicit timing task lies in the nature of the time judgment, which demands more cognitive capacities in terms of working memory and attention in the former than the latter task [31]. The lack of time-related emotion effect in our implicit task thus questions the idea of an automatic acceleration of the internal clock mechanism in emotional contexts. Indeed, this type of automatic speeding up of the internal clock should have produced distortion in time judgment in the implicit task as well as in the explicit task. Several studies have shown the critical role of attention in the processing of threatening stimuli in order to detect possible danger [39,40]. Consequently, if the effects of emotion on time judgment are ultimately due to attention processes rather than to an automatic speeding up of the internal clock, when few attention resources are required to process time as in the implicit task used in our study, then no emotion-related distortion of time should occur. The results obtained in our implicit timing task therefore suggest that attention plays a critical role in the emotion-related distortions of time found in previous studies using explicit timing tasks.

However, our study was the first to examine the effect of emotions on implicit timing in human adults and further experiments are required to be able to conclude. Indeed, several methodological problems could have prevented the emergence of a clear significant emotion effect on implicit timing. Firstly, the emotional stimuli used in our study (emotional facial expressions) were perhaps not sufficiently arousing to induce a significant effect on the implicit timing of action. Other emotional stimuli must therefore be tested using our implicit paradigm. Secondly, in our study, we observed a within-trials influence of emotion on RT. In our experiments, we decided to use a random presentation of faces and interval durations during trial blocks in the emotion session. In this experimental condition, the RT curve for the neutral faces tended to peak at a shorter interval duration in the session involving angry faces than in that involving sad faces. The within-session emotional context might thus have affected RT on neutral trials, thereby limiting the emotion-related differences in RT within the same session. Recently, Gamache et al. [42] showed the importance of discrimination context in time judgment. For instance, when multiple foreperiods (i.e., period of time preceding an interval to be timed) were randomly varied within trial blocks, longer interval durations resulted in longer rather than shorter perceived durations. Further studies are therefore needed to test the effect of emotional context on implicit timing as a function of experimental conditions.

In conclusion, our experiments using an implicit timing task with RT as measure showed an acceleration of the RT in response to angry faces, thus suggesting that action preparation is accelerated in threatening situations. However, our results did not provide a clear demonstration of a significant effect of emotion on the RT, which depended on the temporal interval between the warning and the response signal. This suggests that, in our implicit timing task, the acceleration of motor action was not accompanied by an acceleration of the internal clock mechanism involving in its timing. However, methodological conditions could explain the limited effect of emotion on the timing of action observed in our experimental task, as some of the subsequent statistical analyses suggest. The debate on the nature of the processes involved in emotional effects on time judgment is thus not over.

## Supporting Information

S1 File Reaction time and peak time for Experiment 1 and 2. Mean, standard error and 95% confidence intervals of reaction time and peak time for Experiment 1 and 2.
(XLSX)

## References

[1] Droit-Volet S, Meck WH. How emotions colour our time perception. Trends in Cognitive Sciences. 2007; 1:504–513. 10.1016/j.tics.2007.09.008 18023604

[2] Droit-Volet S, Brunot S, Niedenthal PM. Perception of the duration of emotional events. Cognition and Emotion. 2004; 18:849–858.

[3] Droit-Volet S, Fayolle S, Gil S. Emotion and time perception in children and adults: the effect of task-difficulty. Timing and Time perception. 2016; 1–23.

[4] Fayolle S, Gil S, Droit-Volet S. Fear and Time: Fear Speeds up the Internal Clock. Behavioural Processes. 2015; 120:135–140. 10.1016/j.beproc.2015.09.014 26440426

[5] Tipples J. Negative emotionality influences the effects of emotion on time perception. Emotion. 2008; 8:127–131. 10.1037/1528-3542.8.1.127 18266523

[6] Noulhiane M, Mella N, Samson S, Ragot R, Pouthas V. How emotional auditory stimuli modulate time perception. Emotion. 2007; 7:697–704. 18039036 10.1037/1528-3542.7.4.697

[7] Gil S, Droit-Volet S. Emotional time distortions: The fundamental role of arousal. Cognition and Emotion. 2012; 26,5: 847–862. 10.1080/02699931.2011.625401 22296278

[8] Droit-Volet S, Fayolle S, Lamotte M, Gil S. Time, Emotion and the Embodiment of timing. Timing and time perception, 2013; 0:1–30.

[9] Gibbon J. Scalar expectancy theory and Weber's law in animal timing. Psychological Review. 1977; 84:279–325.

[10] Gibbon J, Church RM, Meck WH. Scalar timing in memory In Gibbon J & Allan L (Eds.), Annals of the New York Academy of Sciences, 423: Timing and time perception. New York: New York Academy of Sciences; 1984; 52–77. 10.1111/j.1749-6632.1984.tb23417.x 6588812

[11] Cheng R-K, Tipples J, Narayanan N-S, Meck WH. Clock Speed as a Window into Dopaminergic Control of Emotion and Time Perception. Timing and Time Perception. 2016; 4:99–122.

[12] Fadok JP, Dickerson TMK, Palmiter RD. Dopamine is necessary for cue-dependent fear conditioning. Journal of Neuroscience. 2009: 29, 36:11089–11097. 10.1523/JNEUROSCI.1616-09.2009 19741115 PMC2759996

[13] Greba Q, Gifkins A, Kokkinidis L. Inhibition of amygdaloid dopamine D2 receptors impairs emotional learning measured with fear-potentiated startle. Brain Research. 2001; 899:218–226. 11311883 10.1016/s0006-8993(01)02243-0

[14] Schirmer A. Emotion. Thousand Oaks, CA, USA: SAGE Publications, Inc. 2015.

[15] Droit-Volet S, Lamotte M, Izaute M. The conscious awareness of time distortions regulates the effect of emotion on the perception of time. Consciousness and Cognition. 2015; 38:155–164. 10.1016/j.concog.2015.02.021 25890486

[16] Coull J, Nobre A. Dossociating explicit timing from temporal expectation with fMRI. Curr Opin Neurobio. 2008; 18, 2:137–144. 10.1016/j.conb.2008.07.011 18692573

[17] Merchant H, Zarco W, Bartolo R, Prado L. The context of temporal processing is represented in the multidimensional relationshops between timing tasks. PlosOne. 2008; 3, 9:e3169 10.1371/journal.pone.0003169 PMC2525837 18779860

[18] Zelaznik HN, Spencer RMC, Ivry RB. Dissociation of explicit and implicit timing in repetitive tapping and drawing movements. J Exp Psychol: Hum Percept Perfom. 2002; 28:575–588. 10.1037//0096-1523.28.3.575 12075889

[19] Niemi P, Näätänen R. Foreperiod and simple reaction time. Psychological Bulletin. 1981; 89:133–162.

[20] Coull J. Neural subtrates of mounting temporal expectation. Plos One Bioloy. 2009; 8:e1000166. 10.1371/journal.pbio.1000166 PMC2711332 19652699

[21] Nobre AC, Correa A, Coull JT. The hazards of time. Curr. Opin. Neurobiol. 2007; 17:465–470. 17709239 10.1016/j.conb.2007.07.006

[22] Vangkilde S, Petersen A, Bundesen C. Temporal expectancy in the context of a theory of visual attention. Phil Trans R Soc B. 2013; 368:20130054 10.1098/rstb.2013.0054 24018716 PMC3758197

[23] Piras F, Coull JT. Implicit, predictive timing draws upon the same scalar represention of time as explicit timing. Plos One. 2011; 6,3:e18203 10.1371/journal.pone.0018203 21464972 PMC3064672

[24] Los SA, van den Heuvel CE. Intentional and unintentional contributions to nonspecific preparation during reaction time foreperiods. J Exp Psychol: Hum. Percept. Perform. 2001; 27:370–386. 11318053 10.1037//0096-1523.27.2.370

[25] Ekman P, Friesen W. Pictures of facial affect. Palo Alto, CA: Consulting Psychologist Press 1976.

[26] Judd CM, McClelland GH. Data analysis: A model comparison approach San Diego, CA: Harcourt Brace Jovanovich 1989.

[27] Izard CE. The psychology of emotions. New York, Plemum Press 1991.

[28] Johnson KA, Burrowes E, Coull JT. Children can implicitly, but not voluntarily, direct attention in time. PLos One. 2015; 10:e0123625 10.1371/journal.pone.0123625 25881188 PMC4399911

[29] Vallesi A, Arbula S, Bernadis P. Functional dissociations in temporal preparation: evidence from dual-task performance. Cognition. 2014; 130, 2:141–151. 10.1016/j.cognition.2013.10.006 24291265

[30] Los SA, Kruijne W, Meeter M. Outlines of a multiple trace theory of temporal preparation. Front. Psychol. 2014; 5:1058 10.3389/fpsyg.2014.01058 25285088 PMC4168672

[31] Droit-Volet S, Coull J. Distinct developmental trajectories for explicit and implicit timing. Journal of Experimental Child Psychology. 2016. 10.1016/j.jecp.2016.05.010 27295205

[32] Naugle KN, Hass CJ, Joyner J, Coombes SA, Janelle CM. Emotional State Affects the Initiation of Forward Gait. Emotion. 2011; 11, 2:267–277. 10.1037/a0022577 21500896

[33] Woods DL, Wyma JM, Yund EW, Herron TJ, Reed B. Factors influencing the latency of simple reaction time. Front. Hum. Neurosci. 2015; 9:131 10.3389/fnhum.2015.00131 25859198 PMC4374455

[34] Bradley MH, Codispoti M, Cuthbert BN, Lang PJ. Emotion and Motivation I: Defensive and Appetitive Reactions in Picture Processing. Emotion. 2001; 1,3:276–298. 12934687

[35] Bradley MM, Codispoti M, Cuthbert BN, Lang PJ. Emotion and motivation I: Defensive and appetitive reactions in picture processing. Emotion. 2001; 1:276–298. 12934687

[36] Frijda NH. The laws of emotion. Mahwah, NJ: Lawrence Erlbaum Associates, Inc. 2007.

[37] Öhman A. Automaticity and the amygdala: Nonconscious responses to emotional faces. Current Directions in Psychological Science. 2002; 11,2:62–66.

[38] Öhman A, Lundqvist D, Esteves F. The face in the crowd revisited: A threat advantage with schematic stimuli. Journal of Personality and Social Psychology. 2001; 80,3:381–396. 11300573 10.1037/0022-3514.80.3.381

[39] Moog K, Bradley B.P. Orienting of attention to threathening facial expressions presented under conditions restricked awareness. Cognitive Emotion. 1999; 13:713–740.

[40] Vuilleumier P. How brains beware: Neural mechanisms of emotional attention. Trends in Cognitive Science. 2005; 9:585–594. 10.1016/j.tics.2005.10.011 16289871

[41] Dimberg U, Thunberg M, Grunedal S. Facial reactions to emotional stimuli: Automatically controlled emotional responses. Cognition and Emotion. 2002; 16:449–471.

[42] Gamache P-L, Zakay D, Laflamme V, Grondin S. Forepeiods’s length and variability influence the perception of breid time intervals. Atten Percept Psychophys. 2015; 77,5:1507–1514. 10.3758/s13414-015-0937-y 26022698

